# Evaluation of in-vitro methods to select effective streptomycetes against toxigenic fusaria

**DOI:** 10.7717/peerj.6905

**Published:** 2019-05-22

**Authors:** Elena Maria Colombo, Cristina Pizzatti, Andrea Kunova, Claudio Gardana, Marco Saracchi, Paolo Cortesi, Matias Pasquali

**Affiliations:** Department of Food, Environmental and Nutritional Science, University of Milan, Milano, Italy

**Keywords:** Fusarium root rot, Antagonism, Seed treatment, Fusarium foot rot, Actinobacteria, *Streptomyces*, Wheat, Biocontrol, Agriculture, Bayesian analysis

## Abstract

Biocontrol microorganisms are emerging as an effective alternative to pesticides. Ideally, biocontrol agents (BCAs) for the control of fungal plant pathogens should be selected by an in vitro method that is high-throughput and is predictive of in planta efficacy, possibly considering environmental factors, and the natural diversity of the pathogen. The purpose of our study was (1) to assess the effects of *Fusarium* strain diversity (*N* = 5) and culture media (*N* = 6) on the identification of biological control activity of *Streptomyces* strains (*N* = 20) against *Fusarium* pathogens of wheat in vitro and (2) to verify the ability of our in vitro screening methods to simulate the activity in planta. Our results indicate that culture media, *Fusarium* strain diversity, and their interactions affect the results of an in vitro selection by dual culture assay. The results obtained on the wheat-based culture media resulted in the highest correlation score (*r* = 0.5) with the in planta root rot (RR) inhibition, suggesting that this in vitro method was the best predictor of in planta performance of streptomycetes against Fusarium RR of wheat assessed as extension of the necrosis on the root. Contrarily, none of the in vitro plate assays using the media tested could appropriately predict the activity of the streptomycetes against Fusarium foot rot symptoms estimated as the necrosis at the crown level. Considering overall data of correlation, the activity in planta cannot be effectively predicted by dual culture plate studies, therefore improved in vitro methods are needed to better mimic the activity of biocontrol strains in natural conditions. This work contributes to setting up laboratory standards for preliminary screening assays of *Streptomyces* BCAs against fungal pathogens.

## Introduction

The use of biocontrol agents (BCAs) against major plant pathogens is becoming a valuable alternative to chemical control of plant diseases and represents an important resource for the future of agriculture (Bardin et al., 2015). The identification of antagonists effective in agricultural environments is essential and requires trustworthy and rapid in vitro selection (Le Cocq et al., 2017) that should be able to simulate as much as possible the conditions that the antagonist will encounter in the field.

*Streptomyces*, a taxonomically wide genus of the phylum Actinobacteria, are gram-positive bacteria ubiquitous in soil as free-living microorganisms and as symbionts of plants, animals, and fungi (reviewed in Seipke, Kaltenpoth & Hutchings, 2012). In most cases, these interactions are positive, as they combine in planta endophytic behavior (Sardi et al., 1992) with the ability to produce an array of specialized metabolites with biological activities (reviewed in Barka et al., 2016). In fact, they have been studied for their antibacterial and antifungal effects (Wei et al., 2017), as well as their plant growth promoting capabilities (reviewed in Viaene et al., 2016), and therefore they can be exploited in agriculture.

*Fusarium graminearum* and *Fusarium culmorum* are the major determinants of severe diseases in cereal crops (Goswami & Kistler, 2004; Scherm et al., 2013), such as fusarium head blight or, under specific climatic conditions, fusarium foot rot and fusarium crown rot (Balmas et al., 2015; Obanor & Chakraborty, 2014). The presence of these pathogens in field affects the yield and the seed quality. In addition, mycotoxins such as trichothecenes type B represent a threat to agricultural production and human health (Maresca, 2013). Three chemotypes can be identified among trichothecene B producing fusaria: (1) producers of nivalenol and acetylated derivatives (NIV chemotype), (2) producers of deoxynivalenol and 3-acetyldeoxynivalenol (3ADON chemotype), and (3) producers of deoxynivalenol and 15-acetyldeoxynivalenol (15ADON chemotype) (Miller et al., 1991). Although the evolutionary history of most of the trichothecene biosynthesis genes is discordant with the species phylogeny, this profile variability can affect the fitness and toxicity of the pathogen population (Ward et al., 2002; Aoki et al., 2012). Diversity of *Fusarium* spp. chemotypes has not been considered in the screening of BCAs to date.

The beneficial effect of Actinobacteria on plant disease suppression is well known (Palaniyandi et al., 2013) and documented by an array of reports on the activity of streptomycetes against toxigenic *Fusarium* spp. (Nourozian, Etebarian & Khodakaramian, 2006; Wang et al., 2013; Jung et al., 2013; Samsudin & Magan, 2016). Even today, after years of research, the lack of appropriate selection methods remains one of the main barriers to the identification of new BCAs (Pliego et al., 2011). Screening for antibiosis is still the most common method adopted in the lab for a high-throughput in vitro screening of biocontrol strains, even though it excludes the identification of other biocontrol mechanisms (Palazzini et al., 2007; Jung et al., 2013). Interestingly, the majority of studies concerning biological control of *Fusarium* spp. used potato dextrose agar medium for assessing the efficacy of BCAs (Faheem et al., 2015; Dubey, Suresh & Singh, 2007; Khan et al., 2006), without taking into account the influence of the culture media on the antifungal activity. Although the final scope of these studies is to withstand pathogen attack in field, selecting effective BCAs directly in the agricultural environment is not always feasible due to inconsistent abiotic and biotic factors encountered in field studies which are also time- and space-consuming. Therefore, in an effort to identify quick and effective methods of selecting *Streptomyces* strains against Fusarium diseases in wheat, we evaluated the effect of *Fusarium* strains, one *F. culmorum* and four *F. graminearum* characterized by different chemotypes, and culture media on the activity of streptomycete strains. Moreover we assessed the correlation of biocontrol activity of streptomyces strains obtained in vitro with their in planta efficacy.

The aims of our work therefore were to identify the most effective medium for in vitro selection of streptomycetes with biocontrol activity to be employed on wheat; test the hypothesis that *Fusarium* strain diversity plays a role in identifying effective BCA strains; and verify if dual culture assays are able to predict the in planta activity of the BCAs.

## Materials and Methods

### Culture media

The antibiosis assays were carried out to test the antifungal activity of 20 *Streptomyces* strains on six different culture media types. PDA1 was prepared using 39 g/L potato dextrose agar (Difco Laboratories, Detroit, MI, USA). One liter of PDA2 was prepared using the extract derived from 200 g of potatoes boiled in distilled water, 20 g glucose (Oxoid Limited, Basingstoke, Hempshire, UK) and 18 g agar (Amresco, Solon, OH, USA). CZY (Czapek-Yeast Extract) contained 35 g/L czapek dox broth, (Difco Laboratories, Detroit, MI, USA), two g/L yeast extract (Difco Laboratories, Detroit, MI, USA), and 15 g/L agar (Amresco, Solon, OH, USA). MMNAG, a modified Minimal Medium (Kieser et al., 2000), was prepared using 4.55 g mannitol (Difco Laboratories, Detroit, MI, USA), 0.5 g KH_2_PO_4_ (Carlo Erba, Cornaredo, Milan, Italy), 0.2 g MgSO_4_ 7H_2_O (Carlo Erba, Cornaredo, Italy), 10 g agar (Amresco, Solon, OH, USA), to which 11.06 g N-acetylglucosamine (Jarrow Formulas, Los Angeles, CA, USA) per liter were added as it has been reported that it may increase secondary metabolite production in *Streptomyces* spp. (Dashti et al., 2017). One liter of wheat meal agar (WMA) was obtained adding the extract derived from 50 g of wheat seeds blended and boiled in distilled water and 15 g agar (Amresco, Solon, OH, USA). One liter of wheat fusarium agar (WFA) was prepared using the extract derived from 50 g of flour boiled in distilled water and 15 g agar (Amresco, Solon, OH, USA). The flour was obtained from wheat seeds inoculated with a mixture of agar-mycelium plugs derived from colonies of *F. culmorum* (FcUK) and *F. graminearum* (PH1) incubated at 24 °C in 250 mL Erlenmeyer flasks in the dark at 100% humidity. The contaminated wheat seeds, after 11-day-incubation, were lyophilized (model Heto-EPD3; Thermo Scientific, San Jose, CA, USA) for 24 h and blended to obtain a homogenous flour. To determine the amount of toxins in the WFA medium, the flour (two g) was extracted with 12 mL of a solution water:CH_3_CN (20:80, v/v), under sonication for 30 min. Then, the mixture was centrifuged at 1,600×*g* for 10 min, and supernatant transferred in a 10 mL flask and the volumes were adjusted by a solution water:CH_3_CN (20:80, v/v). The residues were extracted again as described above. The solutions were centrifuged at 1,000×*g* for 2 min, diluted with water:CH_3_CN (20:80, v/v) and five μL injected into the UHPLC system.

The analysis was carried out on a UHPLC model Acquity (Waters, Milford, MA, USA) coupled with a high resolution fourier transform orbitrap mass spectrometer (model Exactive; Thermo Scientific, San Jose, CA, USA), equipped with a HESI-II probe for ESI and a collision cell (HCD). The operative conditions were as follows: spray voltage −3.5 kV, sheath gas flow-rate 50 (arbitrary units), auxiliary gas flow-rate 15 (arbitrary units), capillary temperature 220 °C, capillary voltage −47.5 V, tube lens −120 V, skimmer −16 V and heater temperature 120 °C. The ion with *m/z* 112.9856 u, corresponding to the formic acid dimer Na adduct [2M+Na−2H]^−^, was used as lock mass.

A 1.9 μm Hypersil Gold C_18_ column (100 × 2.1 mm; Thermo Scientific, Waltham, MA, USA) was used for the separation. The column and samples were maintained at 40 and 20 °C, respectively. The flow-rate was 0.5 mL/min, and the eluents were 0.05% formic acid in water (A) and 0.05% formic acid in CH_3_CN (B). The UHPLC separation was accomplished by the following linear elution gradient: 5–50% B in 6 min and then 50–95% B in 4 min. The acquisition was made in the full-scan mode in the range *m/z* 100–1,000 u, using an isolation window of ±2 ppm. The automatic gain control target, injection time and mass resolution were 1 × 10^6^, 100 ms, and 50 K, respectively. The MS data were processed using Xcalibur software (Thermo Scientific, Waltham, MA, USA). The calibration curves were constructed by dissolving 10 mg of each dried standard in 10 mL methanol. The working solutions were prepared in methanol in the range of 0.02–2 μg/mL for Niv, DON, 4-Ac-Niv, and 3-Ac-DON. All chemicals were purchased from Sigma-Aldrich (St. Louis, MO, USA).

### *Streptomyces* and fungal strains

#### Streptomyces strains

The *Streptomyces* strains were part of a collection of endophytic isolates maintained in the laboratory of Plant Pathology at the Department of Food, Environmental and Nutritional Sciences, University of Milan, Italy (Table 1). Strains were grown on CZY for 2 weeks at 24 °C. Spores were collected adding five mL of a water solution containing 10% sterile glycerol (ICN Biomedicals, Irvine, California, USA) + 0.01% Tween20 (Sigma-Aldrich, St. Louis, MO, USA) to the plate and scraping the surface of the colonies with a sterile loop. The concentration was determined and the spore suspension was stored at −20 °C in small aliquots.

**Table 1:** *Streptomyces* strains used in the study.

Strain code	Source of isolation	Environment of sample collection	Sampling site	Year of sample collection	Closest match as similarity % in EzBioCloud database	Completeness (%)	Genbank accession number
DEF04	*Homo sapiens*	Crypt	S. Fruttuoso (GE, Italy)	1960	99.36%: *Streptomyces marokkonensis*	100%	MK412000
DEF07	*Camellia japonica*	Greenhouse	Arona (NO, Italy)	1988	99.36%: *Streptomyces venetus*	100%	MK412001
DEF09	*Triticum aestivum*	Botanic garden	Milano (Italy)	1990	99.93%: *Streptomyces fulvissimus*	100%	MK412002
DEF10	*Hordeum vulgare* var. *distichum*	Botanic garden	Milano (Italy)	1989	99.86%: *Streptomyces zaomyceticus*	100%	MK412003
DEF13	*Polyporus* sp.	Plane tree	Monza (Italy)	1980	100%: *Streptomyces coelicoflavus*	99.9%	MK412004
DEF14	*Arundo* sp.	Lake shores	Ansedonia (GR, Italy)	1996	99.93%: *Streptomyces fulvissimus*	100%	MK412005
DEF15	*Secale cereale*	Botanic garden	Milano (Italy)	1989	100%: *Streptomyces setonii*	100%	MK412006
DEF16	*Zea mays*	Cultivated field	Cantù (CO, Italy)	1985	99.71%: *Streptomyces albidoflavus*	99.7%	MK412007
DEF19	*Camellia japonica*	Greenhouse	Arona (NO, Italy)	1988	99.37%: *Streptomyces venetus*	100%	MK412008
DEF20	*Carex* sp.	Lake shores	Mergozzo (NO, Italy)	1989	99.37%: *Streptomyces venetus*	100%	MK412009
DEF25	*Homo sapiens*	Crypt	S.Fruttuoso (GE, Italy)	1961	99.36%: *Streptomyces marokkonensis*	100%	MK412010
DEF26	*Triticum aestivum*	Botanic garden	Milano (Italy)	1989	100%: *Streptomyces fulvissimus*	100%	MK412011
DEF35	*Secale cereale*	Botanic garden	Milano (Italy)	1992	99.21%: *Streptomyces neopeptinius*	96.8%	MK412012
DEF38	*Secale cereale*	Botanic garden	Milano (Italy)	1989	100%: *Streptomyces canus*	100%	MK412013
DEF39	*Secale cereale*	Botanic garden	Milano (Italy)	1990	100%: *Streptomyces setonii*	100%	MK412014
DEF41	unknown	Natural environment (savanna)	Canaima (Venezuela)	1993	100%: *Streptomyces costaricanus*	100%	MK412015
DEF43	*Triticum aestivum*	Botanic garden	Milano (Italy)	1989	100%: *Streptomyces costaricanus*	100%	MK412016
DEF44	*Secale cereale*	Botanic garden	Milano (Italy)	1991	99.36%: *Streptomyces marokkonensis*	100%	MK412017
DEF47	unknown	Natural environment (savanna)	Canaima (Venezuela)	1994	100%: *Streptomyces costaricanus*	100%	MK412018
DEF48	*Zea mays*	Cultivated field	Cantù (CO, Italy)	1986	99.36%: *Streptomyces venetus*	100%	MK412019

**Note:**

*Streptomyces* code, the source of their isolation, year, environment and site of sample collection, the percentages of similarity and completeness of the 16S rRNA compared with the EzBioCloud database together with GenBank accession number.

#### Streptomyces identification

DNA from *Streptomyces* isolates DEF04, DEF13, DEF15, DEF25, DEF38, DEF43, DEF44, and DEF47 was extracted following the method described in Sun et al. (2014). A CTAB extraction protocol was used for DEF07, DEF09, DEF10, DEF14, DEF16, DEF19, DEF20, DEF26, DEF35, DEF39, DEF41, DEF48 (Kelly et al., 1998). Analysis of 16S rRNA gene of streptomycetes was conducted using primers 27F (5′-AGAGTTTGATCCTGGCTCAG-3′) and rP2 (5′-ACGGCTACCTTGTTACGACTT-3′). PCR was performed in a total volume of 50 μL, which contained 0.3 μL of GoTaq^®^ DNA Polymerase five U/μL (Promega, Madison, WI, USA), 10 μL of Green GoTaq^®^ Reaction Buffer 5× (Promega, Madison, WI, USA), one μL of 10 mM dNTP (Promega, Madison, WI, USA), one μL of 10 μM primer forward, one μL of 10 μM primer reverse, one μL of template DNA and nuclease free water. The reaction conditions were initial denaturation at 95 °C for 5 min, followed by 35 cycles of denaturation at 95 °C for 20 s, annealing at 56 °C for 30 s and extension at 72 °C for 90 s. A final extension was performed at 72 °C for 7 min. Reaction products were separated by electrophoresis on a 1% agarose gel containing ethidium bromide and visualized under UV light. The PCR products were sequenced in both directions (Eurofinsgenomics, Ebersberg, Germany) using 27F and rP2 primers and two internal primers 16s_p692f (5′-AATTCCTGGTGTAGCGGT-3′) and 16s_p782r (5′-ACCAGGGTATCTAATCCTGT-3′). The sequences were analyzed using EzBioCloud database, which contains quality-controlled 16S rRNA gene and genome sequences (Yoon et al., 2017).

#### Pathogens

Four *F. graminearum* isolates (NRRL 28336 (Pasquali et al., 2016), PH1 (Cuomo et al., 2007), Fg8/1 (Boenisch & Schäfer, 2011), 453 (Pasquali et al., 2013a)) and one *F. culmorum:* FcUK (Pasquali et al., 2013b) were used in this study. They are representatives of different geographical origins and toxin chemotypes (Pasquali & Migheli, 2014). NRRL 28336 and FcUK belong to the 3ADON chemotype, 453 belongs to the NIV chemotype, PH1 and Fg8/1 are characterized by a 15ADON chemotype.

The pathogens were grown on slant agar containing PDA1 and further stored at 5 °C. 4 days before the inoculation for the dual culture assay the strains were transferred on PDA1 dishes at 24 °C.

### Dual culture assay

The influence of culture media on the antifungal activity of the streptomycete strains was evaluated using a dual culture assay in Petri plates (90 mm diameter). The first experiment consisted of a 21 × 5 × 6 factorial with 21 treatments (20 *Streptomyces* and 1 no- *Streptomyces* control), five *Fusarium* strains and six culture media types. The *Streptomyces* strains were inoculated as 10 μL of agar-spore suspension (10^6^ CFU/mL) 3 days before the pathogen to allow their growth as proposed by Kunova et al. (2016). A plug of pathogen agar-mycelium (six mm diameter), taken from the edge of an actively growing *Fusarium* colony, was inoculated upside down in the center of the Petri plate at 25 mm distance from the streptomycete. Three replicate plates were prepared for each strain treatment and plates inoculated only with the pathogen were used as control. Following the inoculation, plates were randomly distributed in an incubator at 24 °C in dark. The radius of *Fusarium* mycelial growth was measured in the direction toward the streptomycete point of inoculation, 3 days after the pathogen application on all six media types. On WMA medium, the incubation was extended in order to assess the antifungal activity up to 7 days.

A repetition of the dual culture experiment was carried out using Fg8/1 strain on WMA medium measuring inhibition rate of the 10 most effective streptomycetes identified in the first experiment.

The antifungal activity was expressed as the percentage of mycelium growth inhibition compared to the control, according to the formula: (*R*_1_−*R*_2_)/*R*_1_ × 100, where *R*_1_ and *R*_2_ were the radius of the pathogen colony in the control and in the presence of the antagonist, respectively.

### In planta assessment of root rot and foot rot severity

To verify the efficacy of the streptomycetes in planta, a modified method from Covarelli et al. (2013) using young plantlets was used. This protocol has been proven to mimic foot and root rot (RR) symptoms development in aged plants (Pasquali et al., 2013b).

A total of 10 *Streptomyces* strains (DEF07, DEF09, DEF14, DEF16, DEF19, DEF20, DEF39, DEF41, DEF47, DEF48), showing higher than 50% inhibitory activity against the target pathogen in dual culture assay, were selected for the seedling assay. The experiment was carried out in glass dishes used as seed trays (diameter 150 mm) sterilized in oven at 160 °C for 2 h. A sterile filter paper was placed in each dish and soaked with 10 mL of sterile water before sowing the seeds. Seeds of wheat “Bandera,” were surface-disinfested in 0.7% sodium hypochlorite for 5 min and then rinsed three times in sterile deionized water using sterile beakers. In a sterile Petri dish, 40 seeds were inoculated with one mL of streptomycete spore suspension (10^7^ CFU/mL). Seeds were left to dry under the laminar flow hood. Control seeds were treated with sterile water. Four dishes were prepared for each treatment containing 10 seeds placed in three rows. The dishes were placed at 5 °C for 24 h to simulate a period of vernalization, then transferred to 20 °C in the dark. After 72 h from treatment with the streptomycete strains, dishes were randomly placed in the growth chamber (21 °C, 16 h photoperiod). Germinating seeds were watered every 2 days with sterile water.

After 4 days of growth, when the roots reached approximately 30 mm, an agar-mycelium plug (six mm diameter) taken from the edge of an actively growing *F. graminearum* Fg8/1 colony, was inoculated upside down on the roots at a 10 mm distance from the seed.

**Figure 1:**
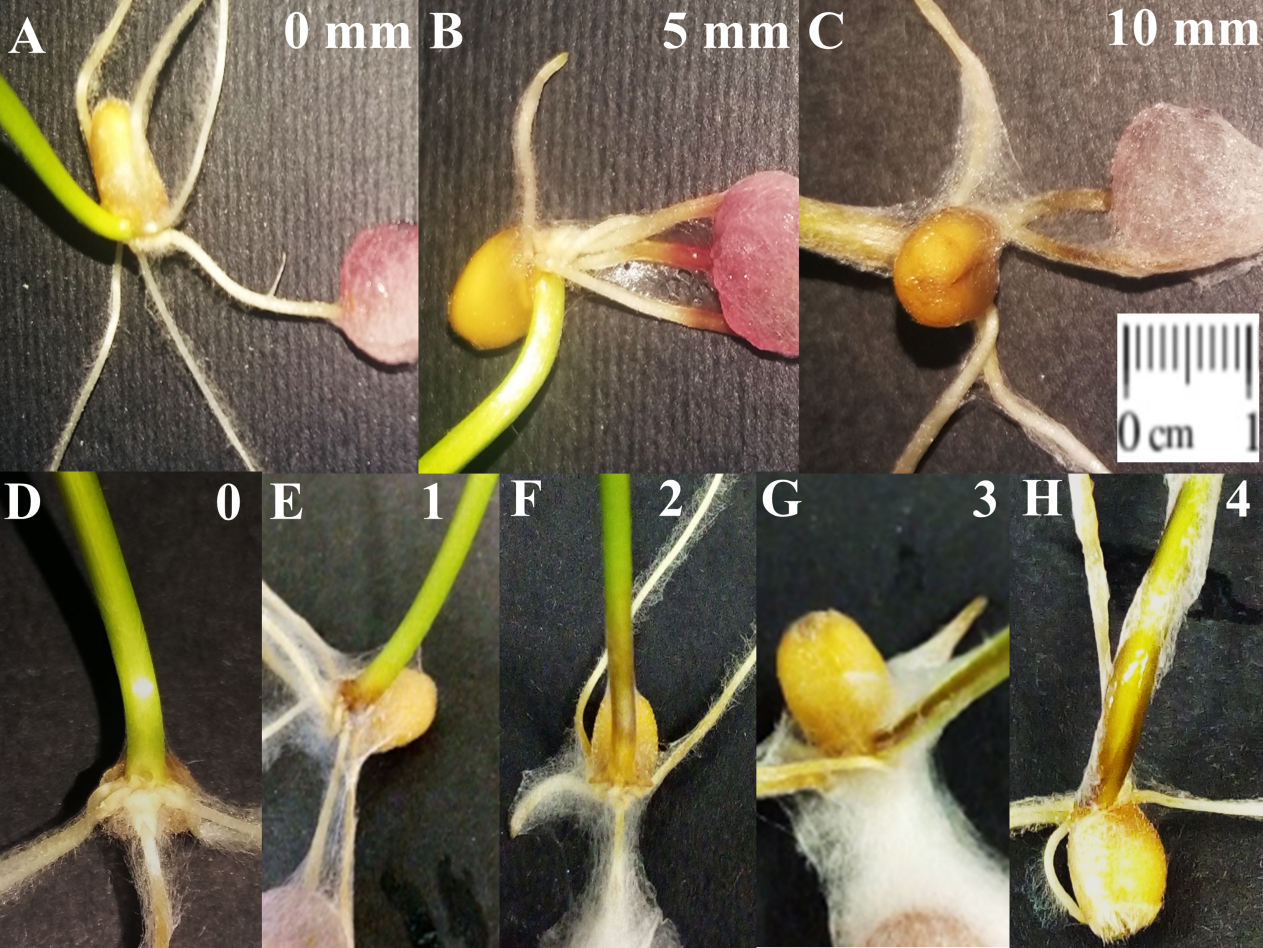
Example of root rot (RR) and foot rot (FR) symptoms on wheat seedlings. (A–C) Length of necrosis of RR measured from the inoculation point (zero, five, or 10 mm from the seed). (D–H) Scale used in FR severity evaluation at the crown level: 0 = symptomless; 1 = slightly necrotic; 2 = moderately necrotic; 3 = severely necrotic; 4 = completely necrotic.

Disease assessments were carried out 4 days after pathogen inoculation, measuring for each plant (*N* = 20 for each treatment) the extension of the necrosis on the root (RR assessment) (Figs. 1A–1C). These data were transformed to percent of necrosis inhibition using the formula (CN−TN)/CN × 100, where CN and TN were the measurements of necrosis on the control and streptomycete treated seedlings, respectively. In addition, 6 day post inoculation (DPI) Foot Rot assessment (FR) was carried out by evaluating the symptoms at the crown level with a zero to four scale (0 = symptomless; 1 = slightly necrotic; 2 = moderately necrotic; 3 = severely necrotic; 4 = completely necrotic) (Figs. 1D–1H). All the observations were used to obtain the disease severity using the subsequent formula:

$$\left[ {\sum {{{\left( {{\rm{Disease \, grade}} \times {\rm{Number \, of \, plants \, in \, each \, grade}}} \right)} \over {\left( {{\rm{Total \, number \, of \, plants}}} \right) \times \left( {{\rm{Highest \, disease \, grade}}} \right)}}} } \right] \times 100$$

The obtained disease severity for each treatment was used to calculate the percentages of protection using the formula (DC−DT)/DC × 100, where DC and DT were the disease severity of the control and the treated seedlings, respectively.

### Statistical analyses

Statistical analyses were performed using R software, version 3.5.1 (R Core Team, 2018, accessed July 2018). R codes and datasets were included in Data S1.

A dual culture assay evaluating the growth inhibition of four *F. graminearum* and one *F. culmorum* strains by a population of streptomycetes was carried out.

To identify the best model able to predict the most active *Streptomyces* strains in vitro considering as parameters media, *Fusarium* strain, and media x *Fusarium* strain, a stepwise forward selection from the simplest linear model -including only one factor- to the most complex one -including all possible interactions was carried out (Table S1). ANOVA was eventually used to test the goodness of the models.

At the same time, model selection using a Bayesian analysis was performed. Consistency of the two approaches was verified. Bayes Factor and the posterior probability of each model were computed with R package “BayesFactor” (Morey & Rouder, 2018).

To assess the repeatability of the dual culture assay, a subpopulation of the ten best performing *Streptomyces* strains was selected. Growth inhibition results of the first and the second experiment (both at 3 DPI and 7 DPI), were compared with a *t*-test. Given the lack of statistical difference between the two experiments, data from the first screening were used to correlate in vitro with in planta results. The objective of the correlation was to understand if dual culture assay carried out with different media could approximate the in planta streptomycete activity.

## Results

### Effect of culture medium and fungal strain diversity on the selection of active *Streptomyces* spp

**Figure 2:**
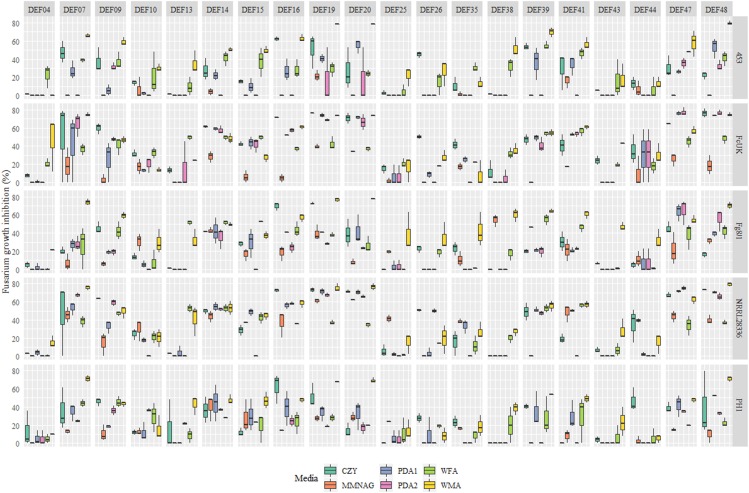
Growth inhibition of five *Fusarium* strains (right labels) by 20 *Streptomyces* strains (top labels) on six media in dual culture assay, measured at 3 DPI (days post inoculation). The six media used for dual culture were: CZY, Czapeck Yeast Agar; MMNAG, Minimal Medium with N-acetylglucosamine; PDA1, Potato Dextrose Agar 1; PDA2, Potato Dextrose Agar 2; WFA, Wheat Fusarium Agar; WMA, Wheat Meal Agar.

A total of 20 *Streptomyces* strains were tested in dual culture assays against five *Fusarium* strains on six culture media types at 3 DPI (Fig. 2). The flour used to prepare the WFA medium had a concentration of DON, 3-AcDON, and NIV of 4.45, 25.8, 0.03 μg/g respectively. Therefore, in a single Petri plate, a total amount of ca. 35 μg of type B trichothecenes was present.

The reproducibility of the plate culture media testing was evaluated by a *t*-test, which confirmed the lack of significant differences between the repeated dual culture assays both at 3 (*P* = 0.9519) and at 7 DPI (*P* = 0.0758) on WMA (raw data in).

To investigate which model could better explain variations occurring in our experiment we compared classical and Bayesian (Table S2) assessments of models. Both approaches confirmed the role of strain, medium and their interaction in explaining the ability of the *Streptomyces* strains to suppress mycelial growth at 3 DPI (Table S3; raw data in Data S1).

Based on the output of the bayesian analysis that suggested the importance of interactions, a multiple regression model was fitted to estimate the effect of each medium, fungal strain and their interactions compared to a selected standard control assay that we defined to be PDA1-Fg8/1 (Table 2, raw data in Data S1). By looking overall at the interactions, PDA2 had similar effects compared to PDA1 confirming that the two media, as expected, are very similar. Also CZY did not significantly differ from PDA1 medium nor did it affect any interaction with fungal species (Table 2). The other media (MMNAG, WFA, WMA) had a significant role in at least some of the interactions. WMA was the most effective medium in highlighting the activity of the *Streptomyces* strains. On this medium, the estimated inhibition for FcUK, NRRL28336, and PH1 strains was significantly different from Fg8/1 (*P* = 0.0001; *P* = 0.0002; *P* = 0.0232) (Table 2).

**Table 2:** Results of multiple regression model on in vitro inhibition of *Fusarium* spp. growth obtained with a population of 20 streptomycetes at 3 DPI (days post inoculation).

Parameter	Coefficient	Std. Error	*t*-value	*P*-value
Intercept	20.16	2.73	7.38	2.35e-13*
453	−3.22	3.86	−0.83	0.40
FcUK	15.10	3.86	3.91	9.53e-05*
NRRL28336	14.71	3.86	3.81	0.00*
PH1	1.02	3.86	0.26	0.79
CZY	5.01	3.86	1.30	0.19
MMNAG	−0.94	3.86	−0.24	0.81
PDA2	−3.54	3.86	−0.92	0.36
WFA	7.81	3.86	2.02	0.04*
WMA	30.73	3.86	7.96	3.09e-15*
453-CZY	2.98	5.46	0.55	0.58
FcUK-CZY	4.28	5.46	0.78	0.43
NRRL28336-CZY	−1.87	5.46	−0.34	0.73
PH1-CZY	−0.36	5.46	−0.07	0.95
453-MMNAG	−13.21	5.46	−2.42	0.01*
FcUK-MMNAG	−22.31	5.46	−4.08	4.62e-05*
NRRL28336-MMNAG	−4.53	5.46	−0.83	0.41
PH1-MMNAG	−7.81	5.46	−1.43	0.15
453-PDA2	−6.64	5.46	−1.21	0.22
FcUK-PDA2	4.71	5.46	0.86	0.39
NRRL28336-PDA2	−0.91	5.46	−0.17	0.87
PH1-PDA2	−1.83	5.46	−0.33	0.74
453-WFA	4.44	5.46	0.81	0.42
FcUK-WFA	−6.72	5.46	−1.23	0.22
NRRL28336-WFA	−12.86	5.46	−2.35	0.02*
PH1-WFA	−8.00	5.46	−1.46	0.14
453-WMA	−1.81	5.46	−0.33	0.74
FcUK-WMA	−21.17	5.46	−3.88	0.00*
NRRL28336-WMA	−20.66	5.46	−3.78	0.00*
PH1-WMA	−12.40	5.46	−2.27	0.02*

**Notes:**

Parameters include fungal strains, media, and their interaction (Model 4, Table S1–S2).The medium PDA1 and the *Fusarium* strain Fg8/1 were set as baseline. Other parameters include the fungal strains 453, FcUK, NRRL28336, PH1, and the media.

CZY, Czapeck Yeast Agar; MMNAG, Minimal Medium with N-acetylglucosamine; PDA2, Potato Dextrose Agar 2; WFA, Wheat Fusarium Agar; WMA, Wheat Meal Agar.

* *P* < 0.05 is considered significant.

There was a significant effect (*P*-value < 0.05) of each *Fusarium* strains (on at least one culture media type) on the ability of streptomycetes to suppress mycelial growth in culture (Table 2).

### Correlation between dual culture assay and in planta activity

Evaluation of potential BCAs using plant-based assays is essential to verify tripartite interactions (pathogen-BCA-plant) that occur in the field. We assessed the disease inhibition on wheat plantlets infected with *F. graminearum* strain Fg8/1 and treated with a subpopulation of streptomycetes (DEF07, DEF09, DEF14, DEF16, DEF19, DEF20, DEF39, DEF41, DEF47, DEF48) on wheat plantlets and compared the results obtained in the dual-plate experiments.

Scoring the protection level granted by each streptomycete on each medium, we assessed which medium best predicted the results obtained on wheat plantlets applying the 10 most effective *Streptomyces* strains.

**Figure 3:**
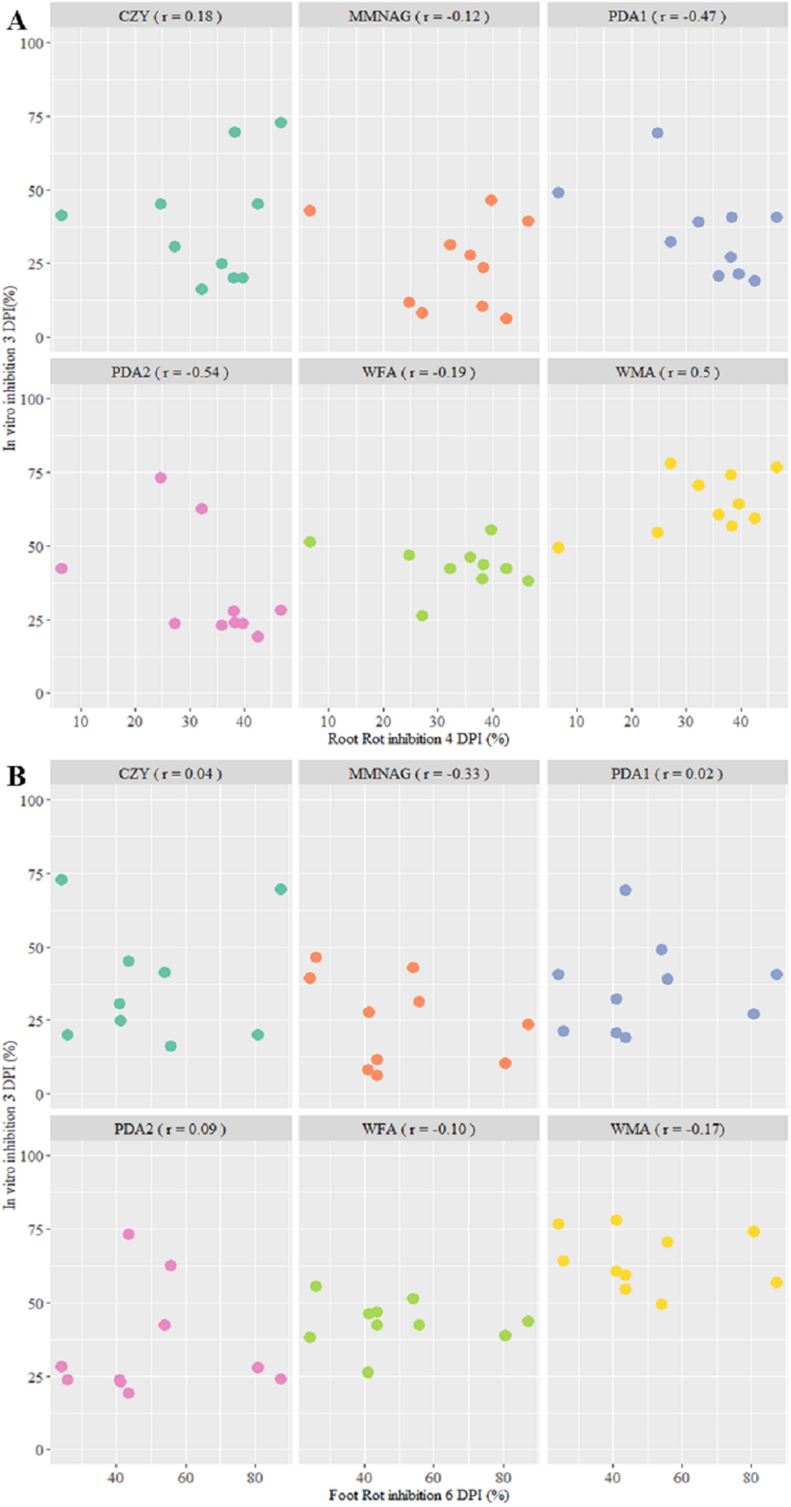
Correlation between in vitro inhibition of *Fusarium graminearum* Fg8/1 growth by 10 streptomycetes on six culture media and in vivo root rot (RR) reduction induced by the same strains (A) and in vivo foot rot (FR) reduction. The streptomycete strains used were DEF07, DEF09, DEF14, DEF16, DEF19, DEF20, DEF39, DEF41, DEF47, and DEF48. The six media used for dual culture were: CZY, Czapeck Yeast Agar; MMNAG, Minimal Medium with N-acetylglucosamine; PDA1, PotatoDextrose Agar 1; PDA2, Potato Dextrose Agar 2; WFA, Wheat Fusarium Agar; WMA, Wheat Meal Agar. Relationship is expressed as correlation value (*r*) in the boxes.

By correlating the protection against wheat RR at 4 days (RR) (Table S4, raw data in Data S1) with the pathogen inhibition in dual culture at 3 days we observed that the majority of the culture media types had low or negative correlation values, therefore not corresponding with the in planta results (Fig. 3A). Only WMA was able to reach a biologically meaningful positive correlation, *r* = 0.5.

The disease assessment on seedlings at 6 days was carried out to evaluate the incidence of a simulated foot rot, estimating the level of necrosis at the crown (Table S4, raw data in Data S1). The results in planta did not correlate significantly with the pathogen inhibition measured at 3 days in the different media plates (Fig. 3B).

We evaluated if the WMA antifungal activity recorded at 7 DPI was able to simulate in planta FR protection (Table S5, raw data in Data S1). No biologically significant correlation between the FR protection and the percentages of inhibition at 7 DPI was observed (*r* = 0.2).

## Discussion

### Selecting the best dual culture assay medium

By assessing multiple media types (*N* = 6) and *Fusarium* targets representing several chemotypes (*N* = 5) we confirmed the complexity of interactions occurring in dual culture assays used for streptomycetes selection as antagonists in agricultural settings (Cao et al., 2004; Boukaew, Chuenchit & Petcharat, 2011; Yekkour et al., 2012; Kunova et al., 2016). Given the potential significant influence of culture medium on the activity of BCAs (Peighamy-Ashnaei et al., 2008) one of our objectives was to verify whether an optimal medium could be identified for selecting *Streptomyces* strains active against toxigenic fusaria. Therefore, in addition to standard media routinely used for dual culture assays (PDA, CZY), we included also newly developed media able to better simulate possible conditions that would be found in the environment. The scope of using a toxin-containing medium was to select strains able to exert their activity also in conditions where the fungus may have already produced the toxins. Interestingly, we observed no difference in the efficacy of individual streptomycetes based on the toxin content in the medium.

Our results suggest that the streptomycetes collection used in our study had similar ability to cope with trichothecene type B. Whether this is due to similar detoxifying capability (Ji, Fan & Zhao, 2016) would require further studies. Although WFA medium was not the most indicative of biocontrol activity, it can be used to test for the sensitivity of BCAs to toxins that may contribute to assess preventively the fitness in real conditions.

Among the other media used, WMA and MMNAG were effective in showing differences between highly active and mildly active *Streptomyces* strains. Taking into consideration all possible combinations of fungal strains and the most effective media WMA, our study shows that antifungal activity of a single streptomycete can vary up to 70%, confirming preliminary observations of Jung et al. (2013), who observed minor effects of different standard media on triggering the ability of streptomycetes to inhibit fungal growth. Our results indicate that more effective media are needed to assess the activity of streptomycetes and BCAs against fusaria.

### Importance of fungal population diversity for BCA identification

The spectrum of activity of an antagonistic strain is often tested against a variety of fungal species (Koch & Löffler, 2009; Al-Askar, Khair & Rashad, 2011; Solans et al., 2016; Kunova et al., 2016). However, the antagonist is rarely tested against a set of diverse representative strains of the pathogen. A positive exception is the recent work of Palazzini et al. (2017), who used a mixture of two *Fusarium* strains to validate the effect in the field. The biological diversity of the target may strongly influence the disease incidence and the response of the pathogen to the BCA treatment (Bardin et al., 2015). In addition, the use of multiple strains can overcome poor outcomes in selection. In our study we included chemotype diversity of *F. graminearum* and *F. culmorum* which are also frequently encountered in different geographical areas as a main cause of Fusarium diseases in wheat. Our results indicate that fungal strain diversity contributes to a lower extent than medium diversity in evidencing streptomycetes activity. In addition, the diversity observed between the two species is comparable to the diversity observed within the *F. graminearum* strains. Nonetheless, the different effects observed among fungal strains are likely due to the complex interactions occurring during the hyphae development and the beginning of sporulation (Hopwood, 1988; Chater, 2006). In particular, different metabolites synthesized by the streptomycetes may play a crucial role in these interactions. Their synthesis can be modulated in different ways (Liu et al., 2013; Abdelmohsen et al., 2015; Antoraz et al., 2015) by fungal compounds that are often strain or group specific (Lind et al., 2017). Therefore, it is possible that the fungal molecules may act as inducers or modulators of streptomycete secondary metabolite production (Fguira et al., 2008), leading to different outcomes in the specific interactions occurring in the plate. Further studies on these metabolic interactions are warranted.

### The complexity of mimicking in planta activity in dual culture assays

The objective of all laboratory approaches to select appropriate BCAs is to better mimic the interactions with the plant that will occur in the field. A large number of successful antagonistic strains selected in laboratories fails when the plant and other environmental factors are taken in consideration (Folman, Postma & Van Veen, 2003). In order to select methodologies able to simulate the interactions between the streptomycetes and the fungus in the natural environment, we decided to verify the correlation of the pathogen inhibition by the biocontrol strain in planta (using a simplified laboratory model) with the in plate co-culture on artificial media. The composition of the culture medium can affect secondary metabolite production leading to a modulation of the synthesis of biocontrol involved molecules (Bode et al., 2002). In particular, the effect of different carbon sources has been considered important for this purpose (Sánchez et al., 2010). Our results indicate that the majority of the media are not good predictors of the streptomycete activity in planta with correlation values that are close to 0 or negative. In general, the presence of the plant can strongly influence the BCAs metabolism resulting in different expression of antibiotic production and this is important to take into account when we want to compare the in vivo and in vitro activity (Haas & Keel, 2003). Interestingly, in our study the only meaningful positive correlation was observed using the WMA, a medium containing wheat metabolites resembling the most in planta conditions. Indeed, plant materials added to the media can elicit the production of specialized metabolites in streptomycetes (Beauséjour et al., 1999).

We assessed both RR and FR in sterile soilless conditions, without considering other environmental interactors that can influence the activity of the BCAs in field. Despite our simplification of the system, we observed only a weak correlation between dual culture and in planta assays (Folman, Postma & Van Veen, 2003). The WMA medium, obtained from wheat seeds, could better predict streptomycetes-mediated protection from RR (*r* = 0.5) but not FR (*r* = 0.2). It is evident that in vitro screening methods play a significant role in our ability to select new BCAs with good chance to succeed in the field. Despite the use of in vitro screening as a common laboratory procedure, even the use of innovative agar media is still not able to simulate the complexity of the plant tissue. Moreover, the signaling during the disease development plays an important role in studying these tripartite interactions. In fact, Beccari, Covarelli & Nicholson (2011) showed that the mode of infection and interaction with the plant of RR and FR follows different pathways. In particular, the severity of FR symptoms does not depend directly from the amount of fungal mycelium growing in the area of infection. Likely, therefore, other biocontrol mechanisms -not only the direct antibiosis- could be elicited. A dual culture assay in plate cannot properly simulate this kind of symptom development.

## Conclusions

Our results showed that the assessment of the antagonistic activity of biocontrol strain is strongly influenced by the adopted method. Many promising antagonistic strains fail when they are tested in planta, due to a lack of appropriate screening procedures (Pliego et al., 2011). Testing the potential biocontrol isolates in field is essential, but not always possible, as it is expensive, time and space consuming and poses procedural challenges given the inconsistency of the abiotic and biotic parameters. While several factors may contribute to diverse activity between the field and the laboratory (Schisler & Slininger, 1997; Folman, Postma & Van Veen, 2003), our results indicate that the selection of the appropriate method of in vitro selection should take into account media and fungal strain diversity, increasing the chance to select truly effective BCAs (Bardin et al., 2015). From the practical point of view, a new single medium selection strategy for *Fusarium* pathogens of wheat would benefit from the use of WMA. Possibly, more than a single *Fusarium* toxigenic strain should be tested.

## Supplemental Information

10.7717/peerj.6905/supp-1 Supplemental Information 1 R Code and tidy datasets used for the analysis. R code (R-script) and datasets used for statistical analysis (Corr_vivo_vitro3dpi.xlsx; Corr_vivo_vitro6dpi.xlsx; Corr_vivo_vitro7dpi.xlsx; Dataset_antibiosistest.xlsx; vivo_vitro_all_3dpi_for graph.xlsx; vivo_vitro_all_6dpi_for graph.xlsx; WMA-FG8_3DPI.xlsx; WMA-Fg8_7DPI.xlsx).

10.7717/peerj.6905/supp-2 Supplemental Information 2 Results of four linear regression models based on *in vitro* inhibition of *Fusarium* spp. growth obtained with a population of 20 streptomycetes at 3 DPI (days post inoculation). The parameters included in the models were media, fungi, both media and fungi and their interaction. Model parameters, degrees of freedom, sum of squares, mean squares, F value and P are indicated for each model. * P<0.05 is considered significant.

10.7717/peerj.6905/supp-3 Supplemental Information 3 Fit of models on *in vitro* inhibition of *Fusarium* spp. growth obtained with a population of 20 streptomycetes 3 DPI (days post inoculation), including as parameters media, fungi, both media and fungi and their interaction. The comparison between models was done with ANOVA (stochastic approach) or with BF (Bayes factor) value for the Bayesian approach. P of ANOVA of the four models including media (model 1), fungi (model 2), fungi plus media (model 3) and also their interaction (model 4) are included; * P<0.05 is considered significant. Bayes Factor (BF) and Posterior probability (P (M|D)) of each model were computed..

10.7717/peerj.6905/supp-4 Supplemental Information 4 Growth inhibition (%) ± Standard Deviation caused by 20 *Streptomyces* strains of 5 *Fusarium* strains at 3 DPI on 6 different media (PDA1, PDA2, CZY, WMA, WFA, MMNAG).

10.7717/peerj.6905/supp-5 Supplemental Information 5 *In vitro* inhibition of *Fusarium graminearum* (Fg8/1) growth by 10 *Streptomyces* strains on six media at 3 DPI data compared to *in vivo* average root rot length (mm) and the percentage of inhibition of root rot and foot rot in plant.

10.7717/peerj.6905/supp-6 Supplemental Information 6 *In vitro* inhibition of *Fusarium graminearum* (Fg8/1) growth by 10 *Streptomyces* strains on WMA medium after 7 DPI and *in vivo* average effect on Foot Rot protection *in planta* at 6 DPI.

## Additional Information and Declarations

### DNA Deposition

The following information was supplied regarding the deposition of DNA sequences: DNA sequences are available from the NCBI Nucleotide Database: MK412000–MK412019.

### Data Availability

The following information was supplied regarding data availability: The raw data are available in a Supplementary File and are associated to the R codes that used them. All the measure of growth in plate and the symptoms on plantlets are reported.
