# Supplementary material for: Evaluation of in-vitro methods to select effective streptomycetes against toxigenic fusaria
Source: PeerJ. 2019 May 22;7:e6905. doi: 10.7717/peerj.6905 (PMC6535041; doi:10.7717/peerj.6905)
Supplement: Supplemental Information 2 — The parameters included in the models were media, fungi, both media and fungi and their interaction. Model parameters, degrees of freedom, sum of squares, mean squares, F value and P are indicated for each model. * P<0.05 is considered significant. [file peerj-07-6905-s002.docx]

|  | Parameters | Degrees of freedom (Df) | Sum of squares (SS) | Mean squares (MS) | F value | *P* (>F) |
| --- | --- | --- | --- | --- | --- | --- |
| Model 1 | Fungi | 4 | 61052 | 15263 | 27.51 | <2e-16* |
| Model 2 | Media | 5 | 158003 | 31601 | 63.07 | <2e-16* |
| Model 3 | Fungi | 4 | 61052 | 15263 | 32.61 | <2e-16* |
|  | Media | 5 | 158003 | 31601 | 67.51 | <2e-16* |
| Model 4 | Fungi | 4 | 61052 | 15263 | 34.11 | <2e-16* |
|  | Media | 5 | 158003 | 31601 | 70.62 | <2e-16* |
|  | Fungi*Media | 20 | 45882 | 2294 | 5.13 | 1.17e-12* |
