# Supplementary material for: Evaluation of in-vitro methods to select effective streptomycetes against toxigenic fusaria
Source: PeerJ. 2019 May 22;7:e6905. doi: 10.7717/peerj.6905 (PMC6535041; doi:10.7717/peerj.6905)
Supplement: Supplemental Information 3 — The comparison between models was done with ANOVA (stochastic approach) or with BF (Bayes factor) value for the Bayesian approach. P of ANOVA of the four models including media (model 1), fungi (model 2), fungi plus media (model 3) and also their interaction (model 4) are included; * P<0.05 is considered significant. Bayes Factor (BF) and Posterior probability (P (M|D)) of each model were computed.. [file peerj-07-6905-s003.docx]

|  | Parameters | P ANOVA | BF | P (M\|D) |
| --- | --- | --- | --- | --- |
| Model 1 | Fungi |  | 8.78e+18 ±0% | 1.54e-71 ±NA% |
| Model 2 | Media | <2.2e-16* | 1.69e+57 ±0% | 2.97e-33 ±NA% |
| Model 3 | Fungi+ Media | <2.2e-16* | 1.66e+80 ±0.75% | 2.93e-10  ±NA% |
| Model 4 | Fungi+ Media+ Fungi*Media | 1.17e-12* | 5.69e+89 ±0.78% | 1 |
