# Supplementary material for: Evaluation of in-vitro methods to select effective streptomycetes against toxigenic fusaria
Source: PeerJ. 2019 May 22;7:e6905. doi: 10.7717/peerj.6905 (PMC6535041; doi:10.7717/peerj.6905)
Supplement: Supplemental Information 5 [file peerj-07-6905-s005.docx]

| ***Streptomyces***  **strain** | **Growth inhibition *in vitro* %** | | | | | | **Root Rot lenght (mm)** | **Root Rot inhibition %** | **Foot Rot Protection %** |
| --- | --- | --- | --- | --- | --- | --- | --- | --- | --- |
|  | **PDA1** | **PDA2** | **CZY** | **MMNAG** | **WFA** | **WMA** |  |  |  |
| **DEF07** | 26.98 **±**  7.27* | 27.84 **±**  7.56 | 20.00 **±**  4.08 | 6.84 **±**  8.94 | 25.88 **±**  23.18 | 74.07 **±**  3.71 | 5.19 **±** 1.80 | 37.22 **±**  21.75 | 24.24 |
| **DEF09** | 19.05 **±**  2.38 | 19.21 **±**  3.60 | 45.10 **±**  6.79 | 6.25 **±**  2.68 | 42.10 **±**  9.94 | 59.26 **±**  3.70 | 4.81 **±** 1.28 | 41.76 **±**  15.45 | 80.86 |
| **DEF14** | 42.06 **±**  14.35 | 35.69 **±**  11.61 | 41.18 **±**  0.00 | 42.86 **±**  3.09 | 51.31 **±**  2.28 | 49.38 **±**  2.14 | 7.84 **±** 1.68 | 5.10 **±**  20.28 | 41.18 |
| **DEF16** | 40.48 **±**  0.00 | 23.92 **±**  5.92 | 69.41 **±**  4.07 | 18.75 **±**  8.61 | 43.42 **±**  8.22 | 56.79 **±**  4.28 | 5.17 **±** 2.28 | 37.47 **±**  27.61 | 43.61 |
| **DEF19** | 40.48 **±**  0.00 | 28.24 **±**  1.18 | 72.55 **±**  1.36 | 39.29 **±**  8.61 | 38.16 **±**  2.28 | 76.54 **±**  2.14 | 4.47 **±** 1.46 | 45.90 **±**  17.70 | 25.93 |
| **DEF20** | 41.49 **±**  16.23 | 23.53 **±**  1.18 | 38.82 **±**  15.43 | 8.04 **±**  3.09 | 26.31 **±**  9.12 | 77.78 **±**  0.00 | 6.11 **±** 2.05 | 26.11 **±**  24.83 | 41.23 |
| **DEF39** | 21.22 **±**  2.07 | 20.00 **±**  6.23 | 20.00 **±**  2.35 | 46.43 **±**  0.00 | 55.26 **±**  6.03 | 64.20 **±**  2.14 | 5.06 **±** 1.26 | 38.82 **±**  15.24 | 43.75 |
| **DEF41** | 20.64 **±**  2.75 | 23.00 **±**  1.73 | 30.20 **±**  10.61 | 21.43 **±**  12.08 | 46.05 **±**  4.56 | 60.49 **±**  4.27 | 5.38 **±** 1.59 | 34.95 **±**  19.20 | 54.17 |
| **DEF47** | 63.49 **±**  9.91 | 64.71 **±**  14.31 | 45.10 **±**  6.79 | 21.43 **±**  17.83 | 38.16 **±**  14.95 | 54.32 **±**  4.28 | 6.31 **±** 1.96 | 23.61 **±**  23.68 | 87.50 |
| **DEF48** | 38.89 **±**  2.75 | 55.29 **±**  12.22 | 16.08 **±**  2.72 | 31.25 **±**  3.09 | 42.10 **±**  8.22 | 70.37 **±**  3.70 | 5.68 **±** 2.33 | 31.21 **±**  28.25 | 55.88 |
| **CONTROL** |  |  |  |  |  |  | 8.26 **±** 1.75 |  |  |

*-mean of mycelium growth inhibition +/- standard deviation
