# Supplementary material for: Evaluation of in-vitro methods to select effective streptomycetes against toxigenic fusaria
Source: PeerJ. 2019 May 22;7:e6905. doi: 10.7717/peerj.6905 (PMC6535041; doi:10.7717/peerj.6905)
Supplement: Supplemental Information 6 [file peerj-07-6905-s006.docx]

| ***Streptomyces* strain** | **Growth inhibition *in vitro* %** | **Foot Rot Protection %** |
| --- | --- | --- |
| DEF07 | 78.82 **±** 2.36 | 24.24 |
| DEF09 | 55.29 **±** 4.08 | 80.86 |
| DEF14 | 58.43 **±** 9.51 | 41.18 |
| DEF16 | 64.70 **±** 2.35 | 43.61 |
| DEF19 | 83.52 **±** 2.36 | 25.93 |
| DEF20 | 83.53 **±** 0.00 | 41.23 |
| DEF39 | 67.05 **±** 4.08 | 43.75 |
| DEF41 | 67.84 **±** 1.36 | 54.17 |
| DEF47 | 64.71 **±** 2.36 | 87.50 |
| DEF48 | 78.04 **±** 1.36 | 55.88 |

*-mean of mycelium growth inhibition +/- standard deviation
